# RNA interference-mediated knockdown of Aurora-B alters the metastatic behavior of A549 cells via modulation of the phosphoinositide 3-kinase/Akt signaling pathway

**DOI:** 10.3892/ol.2014.2464

**Published:** 2014-08-20

**Authors:** LONG DIAN ZHOU, XU XIONG, XIN HUA LONG, ZHI LI LIU, SHAN HU HUANG, WEI ZHANG

**Affiliations:** 1 Department of Orthopedics, The First Affiliated Hospital of Nanchang University, Nanchang, Jiangxi 330006, P.R. China; 2 Department of Respiratory Medicine, The First Affiliated Hospital of Nanchang University, Nanchang, Jiangxi 330006, P.R. China

**Keywords:** non-small cell lung cancer, Aurora B, metastasis, phosphoinositide 3-kinase/Akt signaling pathway

## Abstract

Accumulating evidence has revealed that an elevated expression level of Aurora-B is associated with metastasis in various types of malignant tumor. However, it is currently unclear whether this molecule is involved in non-small lung cancer (NSCLC) metastasis, and the molecular mechanisms associated with Aurora-B and metastasis remain unknown. In the present study, in order to investigate whether Aurora-B is involved in the development and metastasis of NSCLC, the Aurora-B protein expression in NSCLC tissues was detected by immunohistochemistry and its association with metastasis was analyzed. The results revealed that the expression levels of the Aurora-B protein in tissues obtained from NSCLC patients with lymph node metastasis were significantly higher than those without metastatic disease. Furthermore, the effect of Aurora-B inhibition on A549 cell migration and invasion, as well as the activity of the phosphoinositide 3-kinase (PI3K)/Akt signaling pathway was evaluated. Aurora-B was inhibited in the A549 cells using short hairpin RNA, and the cell migration and invasion rates were investigated using wound healing and Transwell invasion assays. In addition, the expression of the main proteins in the PI3K/Akt/nuclear factor-κB (NF-κB) signaling pathway, and matrix metalloproteinase (MMP)-2 and -9 were measured by western blot analysis. The results demonstrated that cell migration and invasion were decreased as a result of silencing Aurora-B. Furthermore, the activity of the PI3K/Akt/NF-κB signaling pathway and the expression of MMP-2 and -9 protein were suppressed by silencing Aurora-B. The results of the present study indicate that the knockdown of Aurora-B suppresses A549 cell invasion and migration via the inhibition of the PI3K/Akt signaling pathway *in vitro* and thus, targeting Aurora-B may present a potential treatment strategy for NSCLC.

## Introduction

Lung cancer is the leading cause of cancer-associated mortality worldwide (1). Approximately 80% of lung cancers are non-small cell lung carcinomas (NSCLC), for which surgery represents the major curative treatment. However, only 30% of NSCLC patients are able to receive surgery. Furthermore, in the majority of cases, the disease is overtly or covertly metastatic on presentation and consequently is currently incurable. Although chemotherapy has been increasingly adopted for advanced NSCLC treatment, the five-year survival rate of NSCLC remains <15% and has not improved in recent years. Therefore, the development of more efficacious targeted therapeutic agents for NSCLC is required.

Aurora B is one of the major protein kinases involved in the execution and fidelity of mitosis. Aurora-B is a member of the chromosomal passenger complex and is involved in numerous mitotic functions, including chromosome-microtubule interactions, sister chromatid cohesion, the spindle-assembly checkpoint mechanism and cytokinesis. Previous studies have shown that Aurora-B is considered to be an important anti-tumor target (2–6). Li *et al* (7) revealed that the downregulation of Aurora-B inhibited proliferation and metastasis, induced G_2_/M phase arrest in clear cell renal cell carcinoma cells and exerted antitumor activity in an SN12C xenograft model. Furthermore, previous studies have shown that the nuclear Aurora-B expression level is significantly associated with metastasis in tumors (8–12). However, whether Aurora-B is involved in NSCLC metastasis remains unclear.

In the present study, the effect of Aurora-B inhibition on A549 cell invasion and migration was investigated *in vitro*. In addition, the effect of silencing Aurora-B on the phosphoinositide 3-kinase (PI3K)/Akt/nuclear factor-κB (NF-κB) signaling pathway was investigated. The aim of the present study was to investigate whether knockdown of Aurora B inhibits A549 cell invasion and migration via downregulation of the PI3K/Akt/NFκB signaling pathway *in vitro*.

## Materials and methods

### Cell cultures

The human NSCLC A549 cell lines (Shanghai Cell Bank, Chinese Academy of Sciences, Shanghai, China) were cultured in Dulbecco’s modified Eagle’s medium (HyClone, Thermo Fisher Scientific, Waltham, MA, USA) with 10% fetal bovine serum (FBS; Sigma-Aldrich, St. Louis, MO, USA) and incubated at 37°C in an atmosphere of 5% CO_2_.

### Patients and specimens

A total of 67 NSCLC tissue samples were obtained from patients who underwent surgery at The First Affiliated Hospital of Nanchang University (Nanchang, China). A total of 28 cases exhibited lymph node metastasis, and 39 cases were identified without lymph node metastasis. The lymph node metastasis survey was performed via histopathological detection of the lymph node. No patient had a history of receiving any prior therapeutic treatment with anti-tumor agents or via radiotherapy. Informed consent was obtained from all patients and the study was approved by the ethics committee of Nanchang University (Nanchang, China).

### Immunohistochemical analysis

Histological sections (4 μm) were stained with hematoxylin and eosin and detected by immunohistochemical analysis, which was performed using the streptavidin-peroxidase procedure. Briefly, antigen retrieval was performed by heating the deparaffinized rehydrated sections in 10 mm citrate buffer (pH 6.0; Abcam, Cambridge, UK) for 20 min, followed by blocking with 10% goat serum (Sigma-Aldrich). Next, sections were incubated overnight at 4°C with the primary rabbit anti-Aurora-B monoclonal antibody (Abcam) at a final dilution of 1:500. For negative controls, sections were incubated with phosphate-buffered saline (PBS; Beijing Solarbio Science & Technology Co., Ltd., Beijing, China) rather than with antibodies. Following three washes with PBS, the sections were incubated with biotinylated secondary monoclonal mouse anti-rabbit antibody (1:1,000; Santa Cruz Biotechnology, Inc., Santa Cruz, CA, USA) for 40 min, followed by incubation with horseradish peroxidase (HRP)-conjugated streptavidin (Beijing Solarbio Science & Technology Co., Ltd.) for 30 min. The sections were then chemiluminescence-stained and counterstained using hematoxylin. The stained sections were subsequently scored by two pathologists that were blinded to the clinicopathological features of the patients. The staining intensity was analyzed (by examining ≥500 cells from five representative areas), the expression level of Aurora-B was evaluated and the intensity scores were recorded as follows: No staining, 0; weak staining, 1; moderate staining, 2; and intense staining, 3. According to the percentage of tumor cells exhibiting a positive expression of Aurora-B, the following percentage scores were used: 0%, score 0; <10%, score 1; 47–50%, score 2; 51–80%, score 3; and 81–100%, score 4. The final score was averaged according to the scores that were determined by the two pathologists according to the percentage scores. The intensity score was then added to the percentage score; a final score of <4 was defined as (−), 4 as (+), 5 as (++) and ≥6 as (+++).

### Recombinant lentivirus-vector (LV) construction and cell transfection

The human mRNA sequence (NM_004217) encoding the Aurora-B protein was obtained from GenBank (http://www.ncbi.nlm.nih.gov/pubmed/). An interfering short hairpin RNA (shRNA) targeting Aurora-B was designed and synthesized, as well as a negative shRNA, which served as a negative control. shRNA sequences were inserted into the LV, GV115 (Aurora-B/LV and negative [Neg]/LV) and transfected into the A549 cells (multiplicity of infection = 20). The transfection efficiency was evaluated using a fluorescence microscope (BX51M, Olympus Corporation, Tokoyo, Japan).

### Quantitative polymerase chain reaction (qPCR) assays

Total RNA from the A549 cells was extracted using TRIzol reagent (Invitrogen Life Technologies, Carlsbad, CA, USA). qPCR was used to detect Aurora-B mRNA expression, with β-actin serving as the endogenous reference gene. All procedures were performed according to the manufacturer’s instructions and the following primers were used: Forward, 5′-AGAAGGAGAACTCCTACCCCT-3′ and reverse, 5′-CGCGTTAAGATGTCGGGTG-3′ for Aurora-B (product length, 202 bp). Six independent experiments were performed over numerous days.

### Western blot analysis

Total protein from the cells was extracted using radio-immunoprecipitation lysis buffer containing 60 lg/ml phenylmethylsulfonyl fluoride [Tiangen Biotech (Beijing) Co., Ltd., Beijing, China]. The protein concentration was determined by Bradford assay (Sigma-Aldrich). Western blot analysis was conducted using the following primary monoclonal anti-human antibodies: Rabbit anti-Aurora-B IgG, (1:200), rabbit anti-PI3K IgG (1:1,000), rabbit anti-phosphorylated (p)Akt IgG (1:800) goat anti-Akt IgG (1:1,000), rabbit anti-NF-κB (p65) IgG (1:400), rabbit anti-matrix metalloproteinase (MMP)-2 (1:1,000) and rabbit anti-MMP-9 IgG (1:1,000), which were all purchased from Abcam and mouse anti-glyceraldehyde 3-phosphate dehydrogenase (1:5,000; Santa Cruz Biotechnology, Inc., Santa Cruz, CA, USA) and the corresponding mouse anti-rabbit, mouse anti-goat and goat anti-mouse monoclonal secondary antibodies (ZSGB-BIO, Beijing, China). The immune complexes were detected using the pro-light Streptavidin-HRP kit (Pierce Biotechnology, Inc., Rockford, IL, USA). Six independent experiments were performed over numerous days.

### Transwell assays

The invasion of A549 cells was measured using the BD BioCoat™ BD Matrigel™ Invasion Chamber (BD Biosciences, Franklin Lakes, NJ, USA) according to the manufacturer’s instructions. The medium in the lower chamber contained 5% fetal calf serum as a source of chemoattractants. The cultures were rinsed with PBS and replaced with fresh quiescent medium alone or containing 10% FBS, following which the cells were incubated at 37°C for 24 h. Cells that had passed through the matrigel-coated membrane were stained using Diff-Quik (Sysmex, Kobe, Japan) and images were captured using a camera (S200, Canon Inc., Tokoyo, Japan) and an inverted microscope (Olympus Corporation). Cell migration was quantified by direct microscopic visualization and counting. The rate of invasion was calculated by counting three fields per membrane and presented as the mean of six independent experiments performed over various days.

### Wound healing assay

Cell migration was analyzed by determining the ability of the cells to move into a cellular space in a two-dimensional *in vitro* wound healing assay. Briefly, cells were grown to confluence in six-well tissue culture plates at a density of ~5×10^6^ cells/well. The cells were then denuded by dragging a rubber policeman (Thermo Fisher Scientific Inc., Rockford, IL, USA) through the center of the plate. Cultures were then rinsed with PBS and replaced with fresh quiescent medium alone or containing 10% FBS, following which the cells were incubated at 37°C for 24 h. Images were captured at 0 and 24 h, and the migrated distance was measured in five independent wound sites per group. Six independent experiments were performed over numerous days.

### Statistical analysis

All data are presented as the mean ± standard deviation. The two independent-samples t-test was used to analyze the difference between Aurora-B protein expression levels in NSCLC patients with and without lymph node metastasis. P<0.05 was considered to indicate a statistically significant difference. All analyses were performed using SPSS version 13.0 (SPSS, Inc., Chicago, IL, USA).

## Results

### Aurora-B protein may be involved in lymph node metastasis in NSCLC

**Figure 1:**
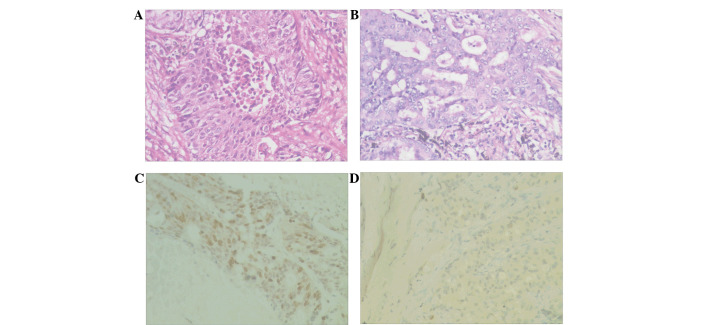
Aurora-B expression in NSCLC patient samples with and without lymph node metastasis (magnification, ×400). Representative images of hematoxylin and eosin-stained NSCLC tissues (A) with and (B) without lymph node metastasis and immunohistochemical staining for Aurora-B in NSCLC tissues (C) with and (D) without lymph node metastasis. NSCLC, non-small cell lung carcinoma.

Aurora-B was expressed in the nucleus (Fig. 1) and the positive expression rate in the samples with metastatic disease was 82.1% (23/28), however, in those without lymph node metastasis the rate was only 43.6% (17/39); the difference was identified to be significant (P<0.05) (data not shown). These results indicated that Aurora-B may be involved in lymph node metastasis in NSCLC.

### Recombinant LV inhibits Aurora-B expression in A549 cells

**Figure 2:**
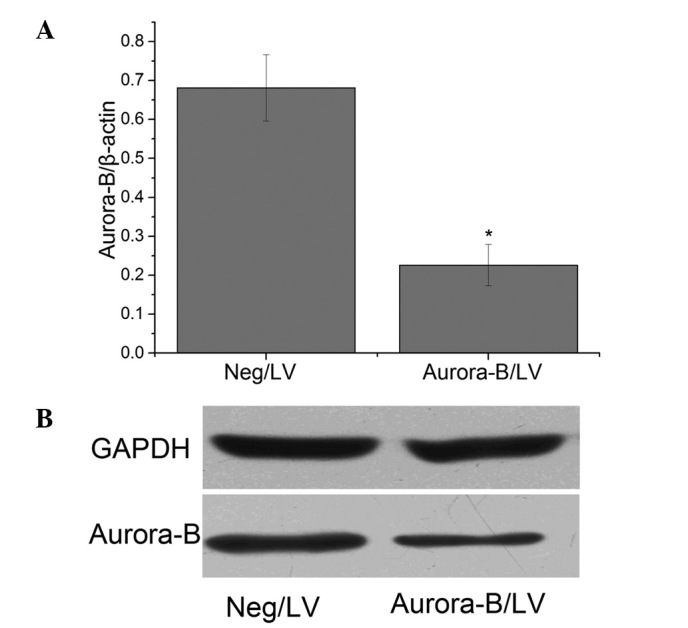
The inhibitory effect of the recombinant LV on the expression level of Aurora-B (A) mRNA (the columns represent the mean of six experiments and the line represents the standard deviation) and (B) protein in A549 cells. ^*^P<0.05 vs. the Neg/LV group. Aurora-B/LV, recombinant lentivirus-vector targeting Aurora-B; Neg/LV, negative lentivirus-vector; GAPDH, glyceraldehyde 3-phosphate dehydrogenase.

A549 cells were transfected with the recombinant LV targeting Aurora-B. qPCR and western blot analysis revealed that the level of Aurora-B protein expression was significantly lower in cells transfected with Aurora-B/LV compared with in those transfected with Neg/LV (Fig. 2).

### Inhibition of Aurora-B suppresses A549 cell migration

**Figure 3:**
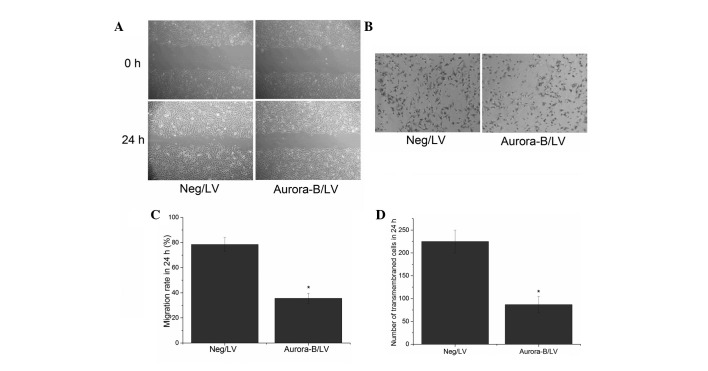
The effect Aurora-B inhibition on A549 cell migration and invasion *in vitro.* Representative images of six experiments of (A) wound healing and (B) Transwell invasion assays are demonstrated for each group. (C) The migration rate of cells was 35.6±3.98% in the Aurora-B/LV transfected group and 78.5±5.66% in the Neg/LV transfected group. (D) The number of transmembrane cells was 87±18 cells per high power field in the Aurora-B/LV transfected group and 225±25 cells per high power field in the Neg/LV transfected group. The columns represent the mean of six experiments and the line demonstrates the standard deviation; ^*^P<0.05 vs. the Neg/LV group. Aurora-B/LV, recombinant lentivirus-vector targeting Aurora-B; Neg/LV, negative lentivirus-vector.

To investigate whether Aurora-B affects cellular migration, an *in vitro* wound healing assay was performed. The results showed that the migration rates of cells transfected with Aurora-B/LV and Neg/LV were 35.6±3.98% and 78.5±5.66%, respectively. The difference was identified to be significant (Fig. 3A and B). These results indicated that Aurora-B inhibition may suppress A549 cell migration *in vitro*.

### Inhibition of Aurora-B suppresses A549 cell invasion

A Transwell assay was performed to evaluate the effect of Aurora-B inhibition on the invasion of A549 cells. The results revealed that the number of transmembrane cells was lower in the cells that had been transfected with Aurora-B/LV (87±18 cells per high power field) when compared with that of the cells transfected with Neg/LV (225±25 cells per high power field [P<0.05]; Fig. 3C and D). These results indicated that the inhibition of Aurora-B may suppress the invasion of A549 cells *in vitro*.

### Silencing Aurora-B inhibits PI3K/Akt/NF-κB signaling in A549 cells

**Figure 4:**
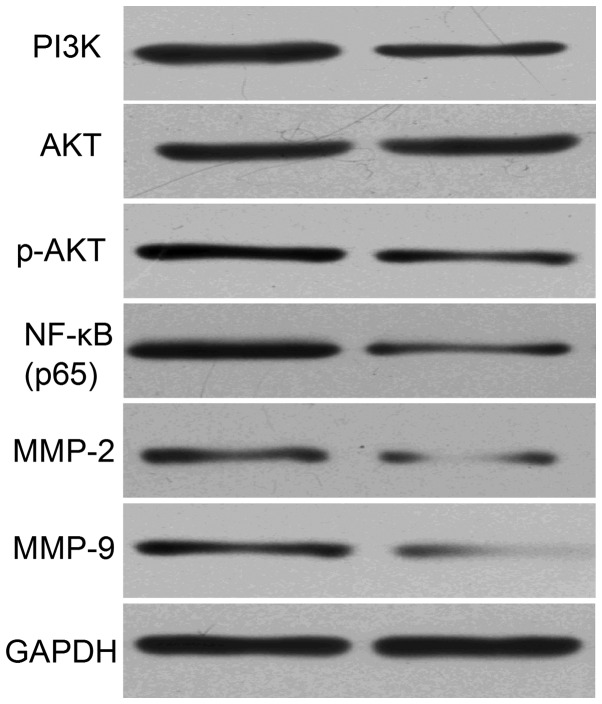
Aurora-B inhibition decreased PI3K/Akt/NF-κB signaling in A549 cells. Right column, cells transfected with Aurora-B/LV; left column, cells transfected with Neg/LV PI3K, phosphoinositide 3-kinase; NF-κB, nuclear factor-κB; p, phosphorylated; MMP, matrix metalloproteinase; GAPDH, glyceraldehyde 3-phosphate dehydrogenase; Neg/LV, negative lentivirus-vector.

In order to investigate the effect of Aurora-B inhibition on the activity of the PI3K/Akt signaling pathway in A549 cells, the expression levels of PI3K, Akt, p-Akt, NF-κB (p65) as well as the MMP-2 and -9 proteins was measured using western blot analysis. The results revealed that the level of PI3K, p-Akt, NF-κB (p65), MMP-2 and -9 protein expression in cells transfected with Aurora-B/LV was significantly lower than that of cells transfected with Neg/LV (Fig. 4). This indicated that the inhibition of Aurora-B may inhibit the activity of the PI3K/Akt/ NF-κB signaling pathway in A549 cells.

## Discussion

Aurora kinases are serine/threonine kinases, which are crucial for cell cycle control and mitosis. Three Aurora kinase family members (A, B and C) have been identified in mammals and are expressed at maximal levels during mitosis. Aurora-B, a component of the chromosome passenger complex, is located on the chromosome arms during prophase, and at the centromeres during the prometaphase and metaphase. Aurora-B subsequently localizes to the midbody during cytokinesis. Previous studies have shown Aurora-B to be overexpressed in numerous types of cancer (9,10,13). In the present study, the expression levels of Aurora-B protein in NSCLC tissues were detected by immunohistochemistry, which revealed that the Aurora-B protein was expressed in the nucleus. Furthermore, the positive expression rate of Aurora-B protein in the NSCLC tissues with lymph node metastasis was significantly higher when compared with the tissue samples without lymph node metastasis. These results are consistent with those reported by Takeshita *et al* (9) and Wang *et al* (14). The results of the present study indicated that Aurora-B may be involved in the development and progression of lymph node metastasis, and may present a novel diagnostic and therapeutic target for NSCLC.

Various studies have revealed that the inhibition of Aurora-B blocked cell proliferation and induced cell apoptosis in a variety of tumors (15–17). These findings highlighted Aurora-B as a potential molecular target for cancer treatment. Notably, recent studies have shown that the upregulation of Aurora-B expression was associated with tumor cell metastasis, and the downregulation of Aurora-B inhibited cell invasion and migration in various tumors (18,19). However, the effect of Aurora-B inhibition in NSCLC malignancies remains to be fully elucidated. In the present study, to investigate the effect of Aurora-B inhibition on NSCLC cell migration and invasion, the recombinant LV targeting Aurora-B was constructed to inhibit Aurora-B expression in A549 cells. Furthermore, the migration and invasion of A549 cells was investigated by wound healing and Transwell assays, and the results revealed that the migration and invasion rate of cells was significantly lower in cells that were transfected with Aurora-B/LV than those that were transfected with Neg/LV. This indicated that the inhibition of Aurora-B may suppress A549 cell migration and invasion *in vitro*.

In addition, the potential molecular mechanisms associated with the inhibition of Aurora-B expression, and A459 cell migration and invasion suppression were analyzed. The role of the PI3K/Akt/NF-κB signaling pathway in tumor cell invasion and migration was investigated (20–25). Long *et al* (26) demonstrated that ZM447439, an inhibitor of Aurora-B, was significantly associated with a decrease in Akt phosphorylation (at Ser473) and a decrease in the phosphorylation of its substrates, glycogen synthase kinase 3-α and -β (at Ser21 and Ser9) in Hep2 cancer cells. Akt is essential for NF-κB activation via the stimulation of the IκB kinase complex, which phosphorylates and inactivates IκB, an inhibitor of NF-κB. Previous studies have demonstrated that NF-κB upregulates MMP-9 (27) and the inhibition of NF-κB was found to downregulate MMP-2 (28). During the development of metastases, cancer cells must degrade the components of the extracellular matrix. MMPs, in particular MMP-2 and -9, are markedly associated with this process due to their capacity to degrade the extracellular matrix, which promotes tumor invasion.

In the present study, PI3K, Akt, p-Akt and NF-κB (p65) protein expression levels were detected by western blot analysis to investigate whether inhibiting Aurora-B led to a decrease in the activity of the PI3K/Akt/NF-κB signaling pathway. The results revealed that the expression levels of the PI3K, p-Akt and NF-κB (p65) proteins were significantly decreased in cells that were transfected with Aurora-B/LV when compared with those in cells that were transfected with Neg/LV. These results indicated that the inhibition of Aurora-B downregulates the PI3K/Akt/NF-κB signaling pathway in NSCLC cells. In addition, western blot analysis was performed to investigate the expression levels of MMP-2 and -9 proteins. It was found that the protein expression levels were reduced as a result of Aurora-B inhibition when compared with the negative control cells, indicating that the inhibition of Aurora-B attenuates the activation of MMP-2 and -9 proteins.

In conclusion, the present study demonstrates that the inhibition of Aurora-B may suppress NSCLC cell invasion and migration via modulation of the PI3K/Akt/NF-κB signaling pathway *in vitro*. This indicates that targeting Aurora-B and the PI3K/Akt/NF-κB signaling pathway may present a potential treatment strategy for NSCLC.
